# Deciphering the Metabolic Changes Associated with Diapause Syndrome and Cold Acclimation in the Two-Spotted Spider Mite *Tetranychus urticae*

**DOI:** 10.1371/journal.pone.0054025

**Published:** 2013-01-17

**Authors:** Samira Khodayari, Saeid Moharramipour, Vanessa Larvor, Kévin Hidalgo, David Renault

**Affiliations:** 1 Department of Entomology, Faculty of Agriculture, Tarbiat Modares University, Tehran, Iran; 2 Université de Rennes 1, UMR CNRS 6553 Ecobio, Rennes, France; U. Kentucky, United States of America

## Abstract

Diapause is a common feature in several arthropod species that are subject to unfavorable growing seasons. The range of environmental cues that trigger the onset and termination of diapause, in addition to associated hormonal, biochemical, and molecular changes, have been studied extensively in recent years; however, such information is only available for a few insect species. Diapause and cold hardening usually occur together in overwintering arthropods, and can be characterized by recording changes to the wealth of molecules present in the tissue, hemolymph, or whole body of organisms. Recent technological advances, such as high throughput screening and quantification of metabolites *via* chromatographic analyses, are able to identify such molecules. In the present work, we examined the survival ability of diapausing and non-diapausing females of the two-spotted spider mite, *Tetranychus urticae*, in the presence (0 or 5°C) or absence of cold acclimation. Furthermore, we examined the metabolic fingerprints of these specimens *via* gas chromatography-mass spectrophotometry (GC-MS). Partial Least Square Discriminant Analysis (PLS-DA) of metabolites revealed that major metabolic variations were related to diapause, indicating in a clear cut-off between diapausing and non-diapausing females, regardless of acclimation state. Signs of metabolic depression were evident in diapausing females, with most amino acids and TCA cycle intermediates being significantly reduced. Out of the 40 accurately quantified metabolites, seven metabolites remained elevated or were accumulated in diapausing mites, *i.e.* cadaverine, gluconolactone, glucose, inositol, maltose, mannitol and sorbitol. The capacity to accumulate winter polyols during cold-acclimation was restricted to diapausing females. We conclude that the induction of increased cold hardiness in this species is associated with the diapause syndrome, rather than being a direct effect of low temperature. Our results provide novel information about biochemical events related to the cold hardening process in the two-spotted spider mite.

## Introduction

Diapause is a common feature in several arthropod species that are subject to seasonally unfavorable growing periods [1], [2]. This genetically programmed developmental response is characterized by a significant slowing (or even cessation) of development, increased body reserves, metabolic suppression, and altered behavior [3]–[6]. The range of environmental cues that trigger the onset and termination of diapause, as well as the associated hormonal, biochemical, and molecular changes, have been extensively studied over the recent years [7]–[12]. However, this information is restricted to a few biological species, with further investigations on non-model arthropod being required to build a comprehensive understanding of diapause phenotype.

Diapause is usually associated with an increase in the degree of resistance to a variety of environmental stresses, which maximize a species’ chances of survival [1]. Even though there is evidence for the independent occurrence of diapause and cold hardiness [13]–[15], both processes are usually linked, occurring in combination in overwintering arthropods [16]–[19]. Diapause represents a prerequisite for subsequent cold hardening in certain insect species, while the non-diapausing species usually exhibit a limited ability for cold acclimation [17]. The enhancement of cold hardiness is a multicomponent process, and involves the biosynthesis of some sugars and polyols. The synthesis of these compounds is promoted by diapause [20]–[22], and is triggered by exposure to low temperatures [23]–[25]. At the early stages of insect diapause, a decline in the expression of enzymes involved in energetic metabolism has been observed [8], together with increased amounts of key enzymes involved in polyols production, such as glucose-6-P dehydrogenase, aldose reductase, and ketose reductase [26]. Then, during diapause development, the amount of accumulated polyols is modulated [26], with subsequent declines in temperature expected to have further effect on the synthesis of these compounds.

Current developments in analytical techniques facilitate the exploration of the functional properties of biochemical and physiological changes associated with diapause syndrome [27], [28]. Among these innovative technological advances, high throughput screening and quantification of metabolites *via* chromatographic analyses may be used to show the quantity of a number of molecules present in the tissue, hemolymph, or whole body of organisms exposed to a given set of conditions [29]. Very few studies used metabolomics, which is a young but rapidly growing discipline in the domain of insect stress physiology [30]–[33], to explore the metabolic fingerprints of diapausing insects [34]. These authors compared the metabolic signatures of diapausing pupae to non-diapausing cold-hardened pupae of the flesh fly *Sarcophaga crassipalpis*. A large increase in glucose and pyruvate was reported [34], which, together with a decline of the molecules of the tricarboxylic acid (TCA) cycle, indicates a decline in aerobic metabolism, with glycolysis being diverted to the pentose phosphate pathway to yield polyols [26]. Subsequently, signs of metabolic depression were confirmed by metabolomics in diapausing specimens of the aphid parasitoid *Praon volucre*, with a drastic reduction in the levels of malate, succinate, and fumarate [28], [35]. However, it remains unknown as to whether the metabolic fingerprints of diapause syndrome are ubiquitous across all insect species.

The two-spotted spider mite, *Tetranychus urticae* Koch (Acari: Tetranychidae), is a well-known plant-feeding pest species worldwide. *Tetranychus urticae* females overwinter in a state of diapause. This facultative diapause is induced by short-day conditions during the period of juvenile development of females [36]. In the present work, we examined the physiological similarities and specificities of cold hardiness and diapause by comparing the metabolic fingerprints of diapausing and non-diapausing females of *T. urticae* in the presence and absence of cold acclimation. In addition to the species’ pest status, we believe that this chill tolerant mite [19], for which the complete genome has been recently published [37], may soon become a biological model for ecophysiological studies. We report the first metabolomics dataset for this species by conducting GC–MS analyses with an uncommon full automated online derivatization process that ensured a complete quality control. We identified the spectrum of metabolites accumulated by cold-hardening in females of *T. urticae*, and examined whether the same metabolites also accumulated in diapausing and cold acclimated females. We hypothesized that (1) diapausing females are characterized by a higher ability to survive exposures at low temperatures compared to non-diapausing females, (2) non-diapausing females exhibit limited cold hardening capacities, and (3) metabolic depression, which is a characteristic of diapausing specimens, results in a large decrease in the concentrations of metabolites involved in the TCA cycle, distinguishing the metabotypes of diapausing and non-diapausing females.

## Materials and Methods

### Collection and Rearing of Mites

Adults of *T. urticae* were originally hand-collected during the summer of 2010 from a bean field near the Tarbiat Modares University in Tehran, Iran (35°44′N, 51°10′E). Adults were directly transferred to the laboratory, and reared on beans under controlled conditions (Light/Dark: 16/8 h; Relative humidity: 70%; Temperature: 27±1°C). Then, more than 30 reared females were sampled at random, and were immediately transferred to detached bean leaves (L/D: 16/8 h; RH: 70%; Temperature: 27±1°C). These females were allowed to lay eggs for 24 h, before being removed from the leaves. Detached bean leaves were placed upside down on a layer of wet cotton in Petri dishes (9 cm in diameter). Each day, water was added to the cotton to keep the arena humid. The lids of the Petri dishes had a 3 cm diameter hole covered with fine nylon mesh to allow ventilation. Hundreds of offspring were then reared until the adult stage, at temperatures of 25°C (L/D: 16/8 h), or 20°C (L/D: 8/16 h), to obtain non-diapausing (ND) and diapausing (D) females, respectively. About 2 weeks was required for ND specimens and 4 weeks for D specimens to reach the adult stage. Subsequently, two batches of newly emerged ND and D females were made. ND and D females from the first batch were directly used in survival experiments, and in metabolic fingerprinting assays. ND and D females from the second batch were randomly transferred to 5°C (NDA5, DA5) or 0°C (NDA0, DA0) for 10 d, at a photoperiod of 12/12 h (L/D) for ND females and 8/16 h (L/D) for D females. In total, there were six experimental conditions: ND, D, NDA5, DA5, NDA0, and DA0 females.

No specific permits were required for the described field studies. The location were the spider mites were sampled are in not privately-owned and not protected in any way. The spider mites represent pest species in Iran, and as such, are not endangered or protected species in any way.

### Survival to Acute Cold Stress

Acclimated (NDA5, NDA0, DA5, and DA0) and non-acclimated (ND and D) females were exposed to subzero temperatures above their supercooling point (SCP) (see [19] for SCP values of female *T. urticae*), to assess mortality caused by cold-related factors other than freezing. Each group of females was transferred to small microtubes (0.5 ml), and was then directly cooled from 25°C to −5, −10, −15, and −20°C at 1°C min^−1^, and kept at these temperatures for 24 h. Four to six replicates were completed for each experimental treatment, with each replicate containing 10 females. The females were subsequently transferred to 25°C (RH: 70%) for recovery. Mortality was determined at 24 h after recovery. Moving specimens, including those that were not able to walk, were considered as alive.

### Effects of Acclimation on the Metabolic Fingerprints of ND and D Mites

Metabolic fingerprinting was conducted in ND, D, NDA5, and DA5 females (4 to 7 replicates per experimental condition, each replicate containing a pool of 100 females). Each sample was weighed (fresh mass) using a Sartorius micro-balance (sensitivity of 0.01 mg), snap-frozen, and stored at −20°C before use in the biochemical assays.

### Sample Preparation and Derivatization

The samples were homogenized in 300 µl of ice-cold (−20°C) methanol-chloroform solution (2∶1) using a tungsten-bead beating apparatus (RetschTM MM301, Retsch GmbH, Haan, Germany) at 25 agitations per second for 1.5 min. Then, 200 µl of ice-cold ultrapure water was added to each sample and then vortexed. After centrifugation at 4,000 g for 5 min at 4°C, 300 µl of upper aqueous phase (which contained polar metabolites) were transferred to new chromatographic glass vials. The vials containing the aliquots were vacuum-dried using a Speed Vac Concentrator (MiVac, Genevac Ltd., Ipswitch, England). The dried aliquots were resuspended in 30 µl of 20 mg. L^−1^ methoxyamine hydrochloride (Sigma-Aldrich, St. Louis, MO, USA) in pyridine, incubated under automatic orbital shaking at 40°C for 90 min. Then, 30 µl of N-methyl-N-(trimethylsilyl) trifluoroacetamide (MSTFA; Sigma, #394866) was added and the derivatization was conducted at 40°C for 45 min under agitation. All the derivatization process was automatized using CTC CombiPal autosampler (GERSTEL GmbH and Co.KG, Mülheim an der Ruhr, Germany) (Fig. S1). Our innovative and uncommon analytical procedure ensures identical derivatization time and process for all samples, and represents a prerequisite for a total quality control.

### GC-MS Analyses

Our GC-MS system consisted of a Trace GC Ultra chromatograph and a Trace DSQII quadrupole mass spectrometer (Thermo Fischer Scientific Inc, Waltham, MA, USA). The injector temperature was held at 250°C. The oven temperature ranged from 70 to 170°C at 5°C.min^−1^, from 170 to 280°C at 7°C.min^−1^, from 280 to 320°C at 15°C.min^−1^, and then the oven remained 4 min at 320°C. We used a 30 m fused silica column (TR5 MS, I.D. 25 mm, 95% dimethyl siloxane, 5% Phenyl Polysilphenylene-siloxane) with helium gas as the carrier at a rate of 1 ml.min^−1^. One microliter of each sample was injected using the split mode (split ratio: 25∶1). We completely randomized the injection order of the samples. The temperature of the ion source was set at 250°C and the MS transfer line at 300°C. Detection was achieved using MS detection in electronic impact (EI). In the present work, we used the selective ion monitoring mode (SIM) (electron energy: −70 eV), ensuring a precise annotation of the detected peaks. Then, we searched for the metabolites that were included in our spectral database (58 pure reference compounds, including the internal standard; Table S1). The peaks were accurately annotated using both their mass spectra (two specific ions) and their retention time. Calibration curves were set using samples consisting of 58 pure reference compounds at levels of 1, 2, 5, 10, 20, 50, 100, 200, 500, 750, 1000, 1500 and 2000 µM. Chromatograms were deconvoluted using XCalibur v2.0.7 software (Thermo Fischer Scientific Inc, Waltham, MA, USA). Metabolite levels were quantified according to their calibration curves.

### Statistical Analyses

Two-way ANOVA with an acceptable significance level of *P*<0.05 was performed for cold tolerance data analysis with SPSS version 16.0 for windows. The correlation between survival and experimental temperature was determined using a binary logistic regression model [38]. Among non-structural carbohydrates, amino acids and organic acids, 47 compounds (out of 58 from our library) were identified in female *T. urticae*, of which 7 were below the quantification limit of the GC-MS (see Table S1). These seven compounds (dopamine, erythritol, galacturonic acid, gentiobiose, saccharose, succinate, and trehalose) were discarded from the multivariate analyses. First, metabolite concentrations, which were expressed as nmoles/mg fresh mass, were log-transformed. Variations in the quantities of total metabolites, total amino acids, and individual metabolites were then examined among the four experimental conditions (D, ND, DA5, NDA5) using an ANOVA with a Tukey *post-hoc* procedure. Second, the cube root transformation of the metabolite quantities was conducted, and the effects of diapause and cold acclimation on the metabolic signatures were investigated using Partial-Least Squares Discriminant Analysis (PLS-DA). Multiple correlation coefficient (R^2^) and cross-validated R^2^ (Q^2^) were used to confirm the predictive power of the fitted model, and the statistical significance of the PLS-DA was also assessed with permutation tests (1000 permutations, *P*<0.001). Variable Importance in Projection (VIP) scores, which are the weighted sums of squares of the PLS loadings, were obtained from the PLS-DA. For the metabolic pathway analysis, the name of the compounds that exhibited significant variations were used, and the significance of the pathway name was assessed with the Holm-Bonferroni method. A heatmap was constructed using Ward’s method as the clustering method and the Pearson correlation as the distance measure. All analyses were conducted using the statistical software of 'R 2.13.0' [39] and MetaboAnalyst (http://www.Metaboanalyst.ca, [40].

## Results

### Survival of Female *Tetranychus urticae* to Acute Cold Stress

**Figure 1:**
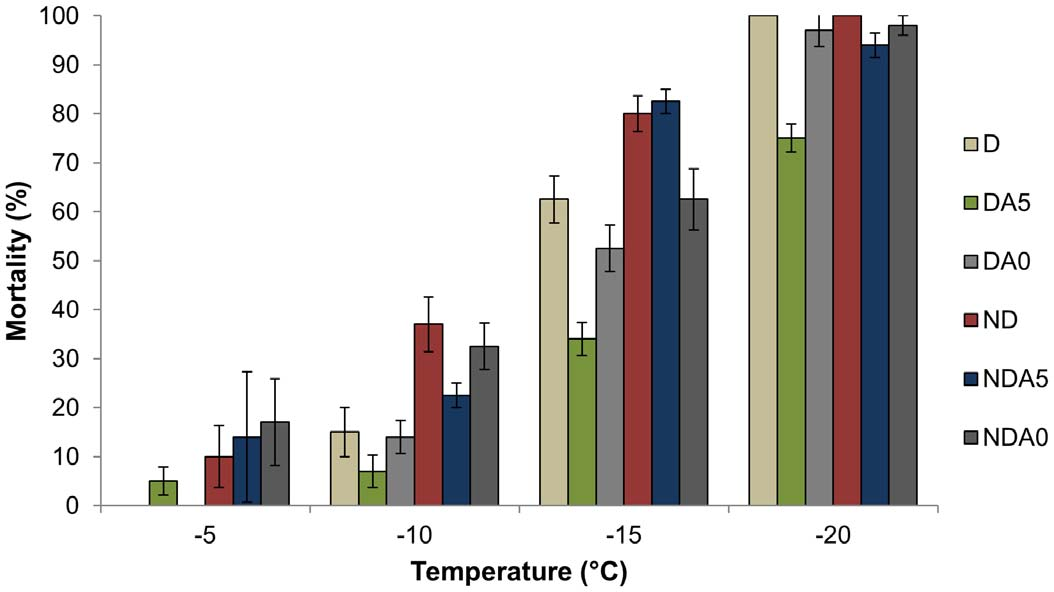
Mortality rate after 24 h of cold exposure. Mortality (mean ± S.E.) was examined in non-diapausing (ND) and diapausing (D) *Tetranychus urticae* females after cold exposures at −5, −10, −15, and −20°C. Non-acclimated mites: ND, D; Mites acclimated at 5°C: NDA5, DA5; Mites acclimated at 0°C: NDA0, DA0.

**Figure 2:**
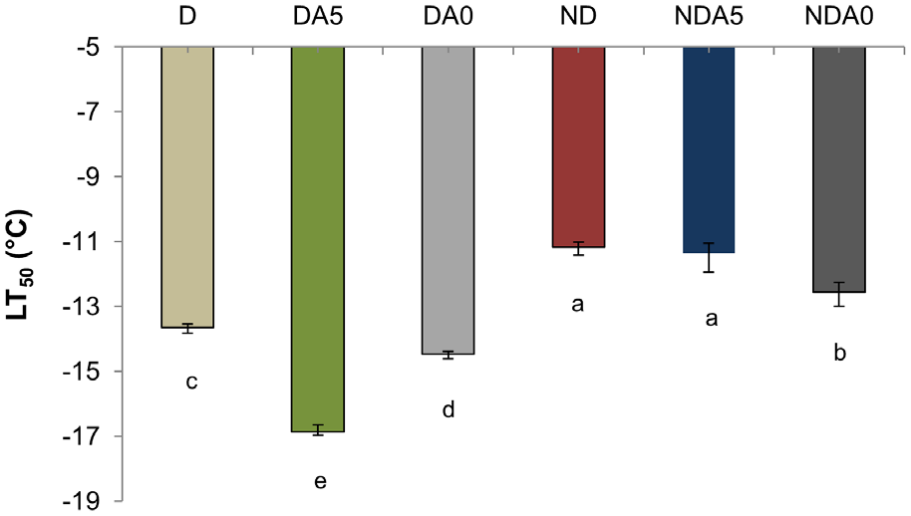
Survival of non-diapausing (ND) and diapausing (D) *Tetranychus urticae* females. Lethal temperature for 50% percent of the population (LT_50_) (± confidence intervals) were determined in non-acclimated mites: ND, D; Mites acclimated at 5°C: NDA5, DA5; Mites acclimated at 0°C: NDA0, DA0. Bars with different letters are significantly different (*P*<0.05).

The mortality rates of females exposed to −5, −10, −15, and −20°C for 24 h are shown in Figure 1. Mortality levels significantly differed among groups (*P*<0.05): DA5 females exhibited the lowest mortality of all experimental conditions. The estimates of temperatures at which 50% of mites died (LT_50_) are shown in Figure 2. Overall, D females were characterized by a lower LT_50_ compared to ND females, with DA5 females exhibiting the highest cold tolerance (*i.e.* the lowest LT_50_).

### Total Amount of Metabolite in Female *Tetranychus urticae*

**Figure 3:**
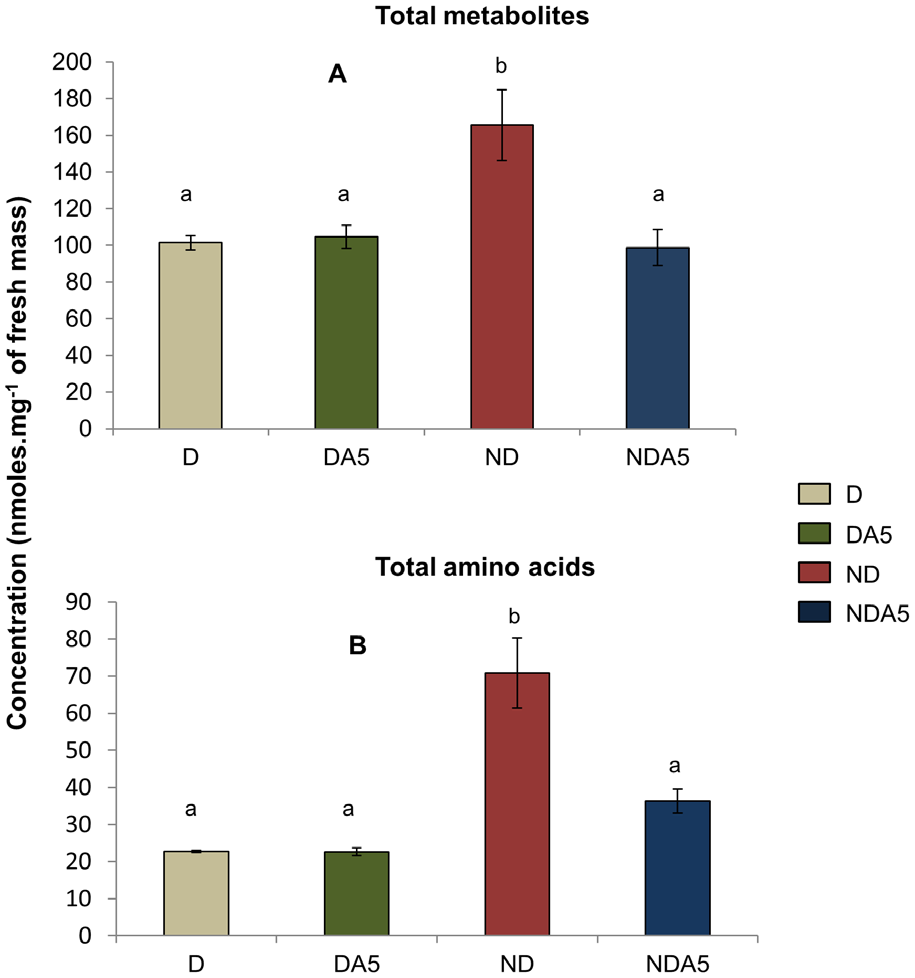
Total metabolite (A) and amino acid (B) content in non-diapausing (ND) and diapausing (D) *Tetranychus urticae* female. Contents are expressed as mean ± S.E. Non-acclimated mites: ND, D; Mites acclimated at 5°C: NDA5, DA5. Bars with different letters are significantly different (*P*<0.05).

A total of 47 metabolites were identified, but only 40 were accurately quantified (Table S1). Of the 40 metabolites, 34 varied significantly among the 4 experimental groups (ND, NDA5, D, DA5; the six non-varying metabolites were: ala, galactose, galactitol, glycerate, triethanolamine, and quinate). Total metabolite and amino acid concentrations significantly differed among the four experimental treatments (Total metabolites: F_3_ = 6.12, *P*<0.01, Fig. 3A; Total amino acids: F_3_ = 14.19, *P*<0.001, Fig. 3B). The Tukey *post-hoc* test (α = 0.05) revealed that ND females had the highest total concentrations (70.9±9.4 nmol.mg^−1^ of fresh mass and 165.5±19.4 nmol.mg^−1^ of fresh mass on average ± SE, for total amino acids and total metabolites, respectively), whereas there was no significant difference for the other acclimated females of *T. urticae*, which had lower concentrations of these compounds (*ca.* 27 and 100 nmol.mg^−1^ of fresh mass for total amino acids and total metabolites, respectively) (Fig. 3A, 3B).

### Effects of Acclimation on the Metabolic Fingerprints of ND and D Mites

**Figure 4:**
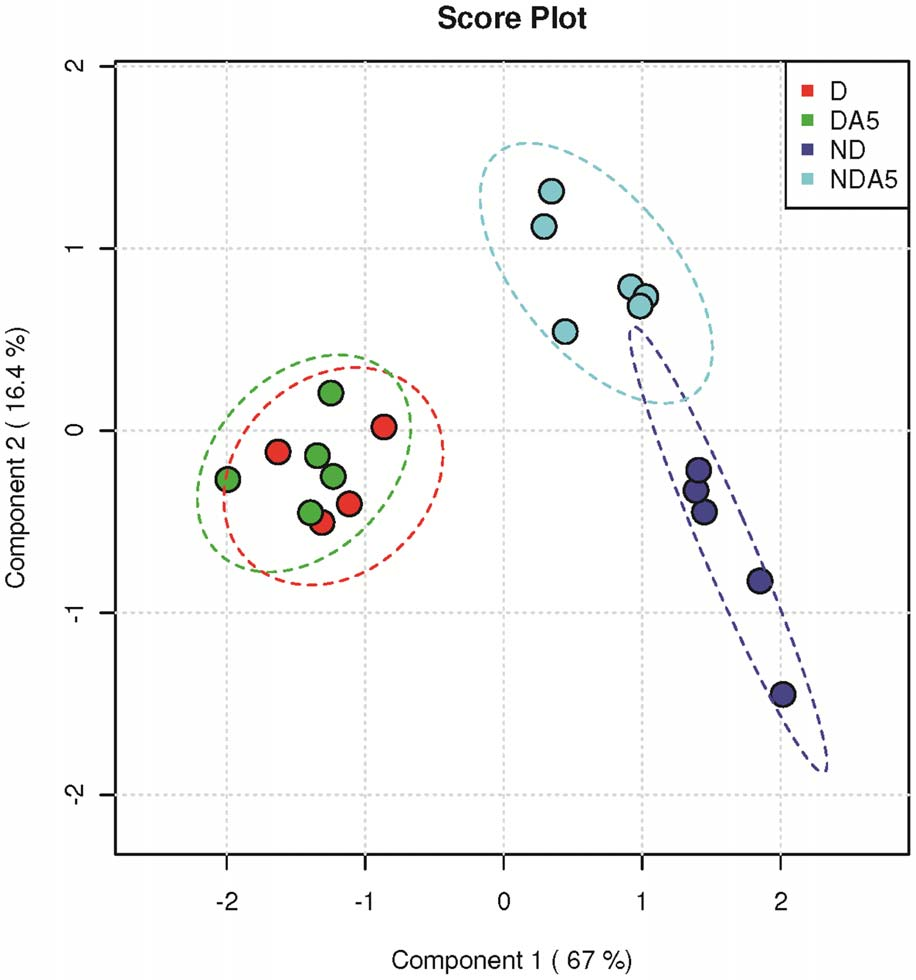
Multivariate analysis (PLS-DA) on metabolomic data. A: sample projection (4–7 samples) onto the first PLS-DA discriminant plane of non-diapausing (ND) and diapausing (D) *Tetranychus urticae* females, that were non-acclimated (ND, D) and acclimated at 5°C (NDA5, DA5). B: the variable importance plot shows the metabolites that contributed the most to the first axis (based on VIP scores).

**Figure 5:**
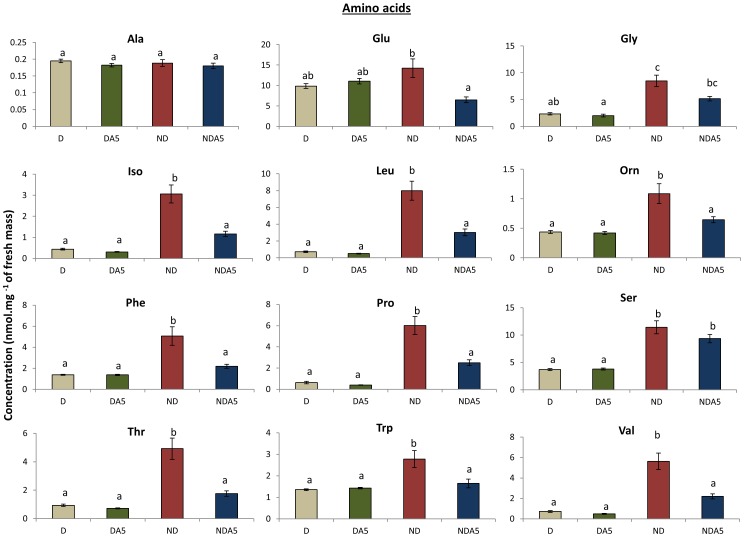
Metabolite content of non-diapausing (ND) and diapausing (D) *Tetranychus urticae* females. Metabolite content is expressed as mean ± S.E. Non-acclimated mites: ND, D; Mites acclimated at 5°C: NDA5, DA5. Bars with different letters are significantly different (*P*<0.05).

Class separation by the 34 discriminant variables was investigated in a PLS-DA (Fig. 4A). The first (LD1) and second (LD2) Linear Discriminant axes accounted for 67.0 and 16.4% of total inertia, respectively. A clear separation among groups was found on LD1, which opposed ND specimens to D ones, regardless of acclimation treatment (Fig. 4A). The VIP scores showed that separation on LD1 was mainly due to malate and glucose (Fig. 4B). D and DA5 females were characterized by high amounts of cadaverine, gluconolactone, inositol, glucose, maltose, mannitol and sorbitol, and lower amounts of almost all remaining compounds (Fig. 4B and 5). Based on the seven listed metabolites, metabolite pathway analysis revealed that only the galactose pathway was significantly enriched (Holm-Bonferroni test, *P*<0.05) in D specimens.

**Figure 6:**
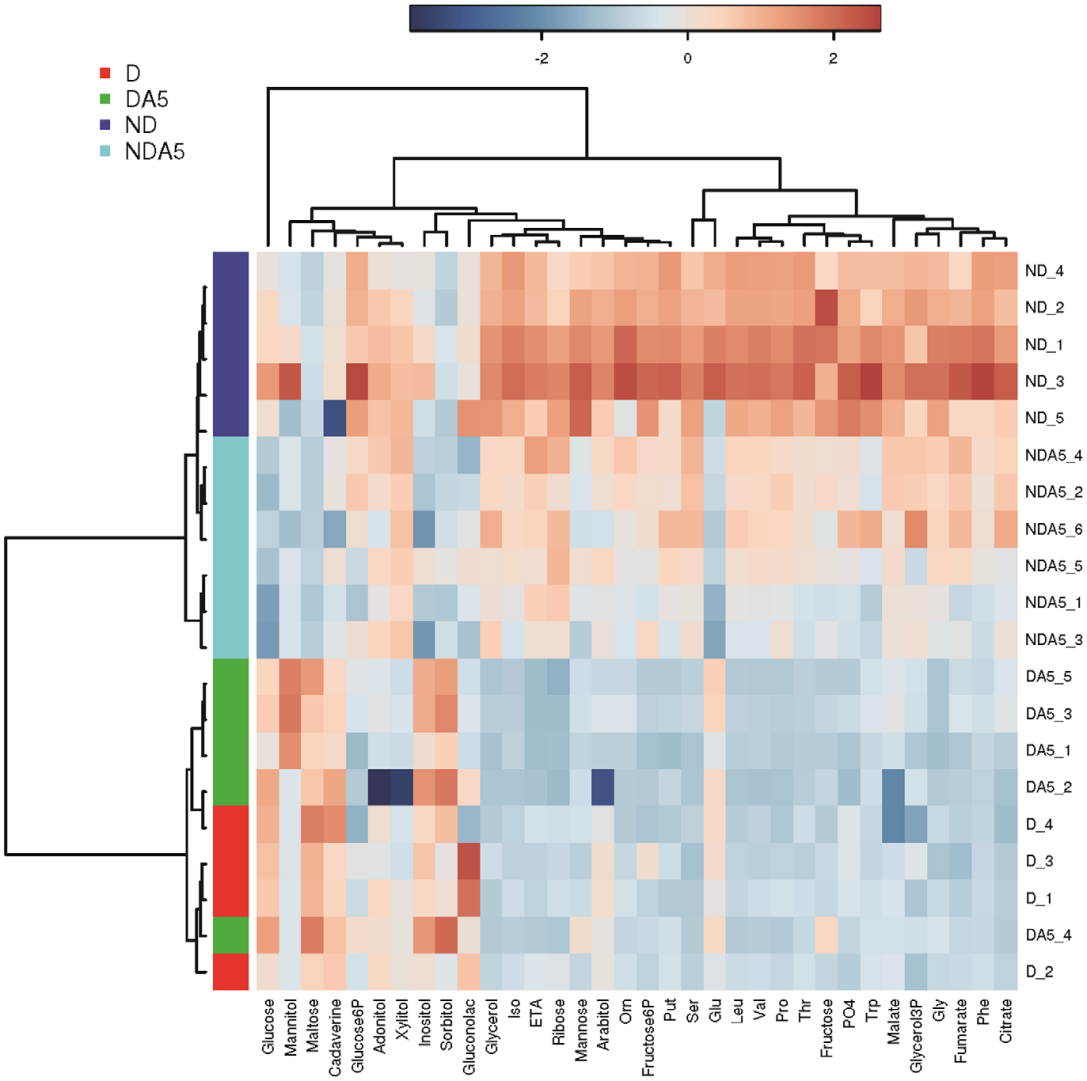
Heatmap of the entire metabolomics dataset. The colors represent the cube root changes of each metabolite relative to the mean control level. Individual samples (horizontal axis) and compounds (vertical axis) are separated using hierarchical clustering (Ward’s algorithm), with the dendrogram being scaled to represent the distance between each branch (distance measure: Pearson’s correlation). The clusters containing D and DA5 females are highlighted red and green, respectively. The clusters containing ND and NDA5 females are highlighted in dark and light blue, respectively.

D and DA5 groups continued to overlap on the 2D-projection of the PLS-DA, and ND and NDA5 females were secondarily separated on LD2 (Fig. 4A). A heatmap diagram showed that the metabolic signatures of D and DA5 females were similar, with the levels of a number of metabolites being clearly down regulated (Fig. 6). Distinct metabolic signatures were more apparent between ND and NDA5, as depicted by the dendrogram (Fig. 6).

## Discussion

### Diapause Enhances the Level of Cold Hardiness of *Tetranychus urticae* Females

In the present study, the physiological basis of cold tolerance in female *T. urticae* spider mites was examined. Congruent with our previous study [19], diapause significantly increased the level of cold hardiness in this species: DA5 females exhibited the highest survival ability of all experimental conditions. As the ND and D females were reared at slightly different temperatures (25 *versus* 20°C) before being cold acclimated, this procedure may have slightly increased the differences in thermal tolerance between ND and D mites. Supporting our hypotheses, acclimation had a slight beneficial effect on ND specimens, with LT_50_ ranging from *ca.* −11 to *ca.* −12°C, depending on the acclimation treatment. Conversely, LT_50_ ranged from −13.5 to −17°C in D mites. The beneficial effects of acclimation on the survival of *T. urticae* ND females were more visible when the duration of cold-exposure was significantly shorter [19]. By increasing the duration of pre exposure to acute thermal conditions before assessing the survival ability of mites, chill injuries may have accumulated to such an extent that we exceeded the point at which the effects of acclimation could be significantly visible in ND mites. In contrast the opposite results were obtained for diapausing mites, with survival ability being significantly improved when females were acclimated. Diapause is usually associated with enhanced cold tolerance in insects [41]; however, diapausing specimens usually become more cold-hardy when subject to cold exposure [17]. In support of the published literature, our results indicate that additional physiological changes probably occurred during cold acclimation in D females, and better protected mites by enhancing their level of cold tolerance.

### Metabolic Signatures Depicted the Depressed Metabolism of Diapausing Mites

As large numbers of specimens were required for each biological replicate in the metabolic fingerprinting analyses, we focused on NDA5 and DA5 specimens. Data about the cold hardiness of the two-spotted spider mite remain limited, with the current study providing the first report about the metabolome of this species. Hence, our preliminary research about the plastic rearrangement of the metabolic networks of D and ND females provides baseline information regarding the biochemical adjustments that occur during diapause and acclimation in this species.

Distinct metabolic signatures were observed among mites exposed to distinct experimental conditions, indicating that the metabolic networks were rearranged to maintain metabolic homeostasis and performance of the organisms [42]. Diapause had the strongest effect on the metabolic signature of female *T. urticae*. In these specimens, we observed a down-regulation in the quantity of several of the measured compounds, including most amino acids and some sugars, supporting that reported for the aphid parasitoid *Praon volucre* [28]. Several of these metabolic changes arise in relation to diapause syndrome, which is characterized by strong metabolic depression and developmental arrest [28], [34]. The entry of D females into a hypometabolic state, which was depicted by lower amounts of TCA cycle intermediates compared to ND females, reduces the energetic demand at the level of the whole organism, and has been considered as an adaptive energy-saving strategy [43]–[44]. Developmental arrest also contributed towards explaining the significant reduction of most amino acids, as these biochemical compounds represent the elementary components of many biological structures, including cuticle formation in arthropods and vitellogenesis in mature females.

Most amino acids were down regulated after diapause induction, with only the levels of alanine and glutamate amino acids remaining high in D females of *T. urticae*. It is not clear to what extent these amino acids may be involved in the response mosaics that assist the winter survival strategy of two-spotted spider mite females; however, some authors have reported their importance in diapausing and cold acclimated insects [45]–[47]. For instance, a lower acclimation temperature, together with diapause, increased the amount of alanine in *Mamestra brassicae* L. [48], which may have occurred as a result of the low aerobic conditions experienced by the insects during diapause, rather than temperature or diapause. Furthermore, an increase in alanine and glutamate was reported in the fat body of *Osmoderma eremicola* (Knoch) larvae during freezing to −8°C for 96 h [49]. Proline, which is a well-known energy source and cryoprotectant in some insects [50], decreased after diapause induction and cold acclimation. It seems that this amino acid is used as an energy source to fuel aerobic metabolism, and is converted to alanine during diapause. Proline levels were also reduced during diapause in the embryo of *Bombyx mori* (L.) [51], and in chilled diapausing larvae of the grass stem borer, *Enosima leucotaeniella* (Ragonot) [47].

While the metabolic fingerprints of D and DA5 females overlapped, the metabolic signatures of ND and NDA5 females were clearly distinguished. Combined with the survival data, our results demonstrated that diapause is a prerequisite for enhanced cold tolerance in *T. urticae* females. No polyols were accumulated in NDA5 females, and the reduction of the whole metabolome, including glucose and TCA cycle intermediates, may have only indicated a general decrease in temperature-dependent aerobic activities.

### The Galactose Pathway was Boosted for the Production of Polyols in Diapausing Females

While the levels of most metabolites were reduced in D females, the concentrations of glucose remained stable, regardless of experimental condition. Glucose represented 50% of the total metabolite pool in D mites *versus* 30% in ND individuals. Furthermore, the high levels of glucose, maltose, inositol, mannitol, sorbitol, and gluconolactone indicated that the pentose phosphate pathway was boosted in D females. As far as we know, earthworms and amphibians are the only animal groups that utilize glucose as their primary cryoprotectant [52], [53], although this osmolyte may have some potential disadvantages [54]. In the fruit fly *Drosophila melanogaster*, even though glucose was hypothesized to have a role in the rapid cold hardening response [55], the quantity of glucose was not related to the level of basal thermo-tolerance of flies [56].

Elevated quantities of glucose are a common feature in diapausing arthropods [28,34,57,58, this study]. Consistent with the high levels of glucose in D mites, we also found an accumulation of gluconolactone. Gluconolactone represents an oxidized derivative of glucose, and might serve as an important scavenger of free radicals in diapausing mites. In addition, gluconolactone is the precursor of gluconolactone-6-phosphate in the pentose phosphate pathway, which is typically elicited in insects exposed to harsh environmental conditions [26]. The pentose phosphate pathway is a major reductant source in the form of NADPH [59], which may be re oxidized for the synthesis of sorbitol. A 20-fold increase in the activity of aldose reductase, the NADP(H)-dependent enzyme catalyzing the transformation of glucose to sorbitol [60], has already been demonstrated in diapausing specimens of the red firebug *Pyrrhocoris apterus* [26]. In the present study, sorbitol increased two-fold in D *T. urticae* females, with a 4-fold increase being found in DA5 individuals. Sorbitol is a well-known cryoprotectant in insects [61], [62], which is assumed to contribute to survival at low temperatures in a similar manner to glycerol.

Other than sorbitol, mannitol, and, to a lesser extent, inositol, were specifically accumulated in diapausing specimens, supporting that found in the aphid parasitoid *P. volucre* [28]. Diapausing and cold-acclimated arthropods are usually characterized by a partial dehydration, and thus, slightly higher concentrations of these compounds may have been reported in female *T. urticae* if metabolite concentrations had been expressed per mg dry mass. Accumulations of mannitol [63]–[65] and inositol [57], [58], [66] have also been reported in some other arthropod species. The synthesis of polyols is assumed to coincide with diapause in many insect species [26], [67], with these compounds playing a significant role in overwintering success. While polyols had no significant effect on supercooling ability in diapausing *P. apterus*, polyol (sorbitol and ribitol) accumulation resulted in a 10-fold increase in survival duration, possibly because of the preferential exclusion of solutes from macromolecules [64], [68].

Interestingly, glycerol, which is the most commonly known polyol that is produced in cold-hardy insects [69], [70], was consistently low in *T. urticae*. The highest amounts of glycerol were found in ND females, which decreased after cold acclimation and diapause induction. A similar result was reported for *Pieris brassicae* (L) [71]. Hence, it is thus unlikely that glycerol played a role in the thermotolerance of the two-spotted spider mite in the current study.

### Conclusion

To conclude, our study confirmed that the induction of increased cold hardiness is associated with diapause in *T. urticae* females. Non-diapausing females were characterized by a limited ability to become cold acclimated, which confirmed that diapause is a prerequisite for enhanced cold tolerance in species overwintering in a diapause state. Our metabolic fingerprints were consistent with investigations on enzymatic activities conducted in a previous study [26]. While the levels of most compounds were down regulated in D females, glucose levels remained elevated, together with the high concentrations of gluconolactone, which may be involved in the formation of reducing equivalents that may be involved in the production and accumulation of polyols (*i.e.,* sorbitol, mannitol, and inositol).

## Supporting Information

Figure S1 **A picture of the GC-MS system.**, which consists of a Trace GC Ultra chromatograph, a Trace DSQII quadrupole mass spectrometer (Thermo Fischer Scientific Inc, Waltham, MA, USA), and CTC CombiPal autosampler (GERSTEL GmbH and Co.KG, Mülheim an der Ruhr, Germany) that automatized all the derivatization process.
(JPG)

Table S1 **List of metabolites identified in females of** ***Tretranychus urticae*** **by GC/MS.** Metabolites were classified according to eight categories: amino acids, polyols, sugars, intermediates of the citric acid cycle, other metabolites, non-quantified metabolites, internal standard and metabolites not found in any treatment. Non-quantified metabolites: the metabolites were detected but their concentrations were below the quantification limit (<QL) of the GC-MS. The minimal QL above which the metabolites can be accurately quantified is given in µM for each compound. Minimum and maximum concentrations (nmoles/mg fresh mass) detected among all treatments are also shown.
(XLS)
