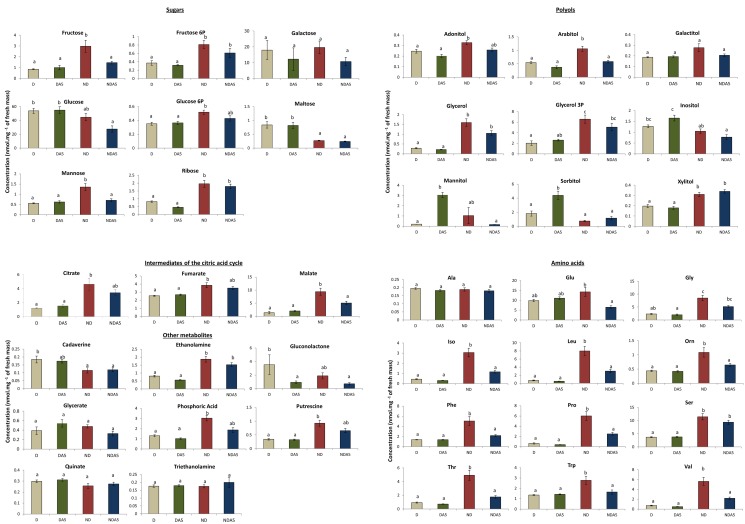# Correction: Deciphering the Metabolic Changes Associated with Diapause Syndrome and Cold Acclimation in the Two-Spotted Spider Mite *Tetranychus urticae*

**DOI:** 10.1371/annotation/e165a24a-a1f6-4fd6-8af9-693ace8748c3

**Published:** 2013-03-01

**Authors:** Samira Khodayari, Saeid Moharramipour, Vanessa Larvor, Kévin Hidalgo, David Renault

There is information missing in Figures 4 and 5. Please view the correct versions of Figures 4 and 5 here:

Figure 4: 

**Figure:**
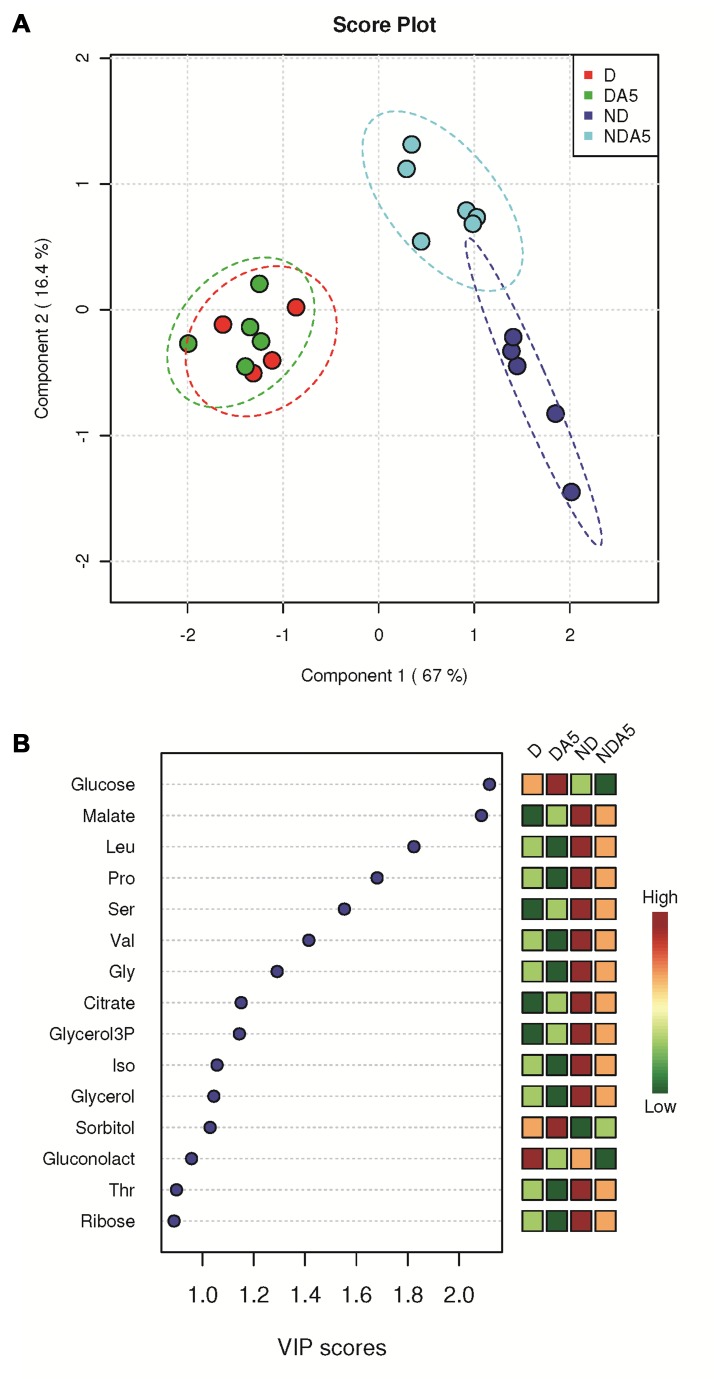


Figure 5: 

**Figure:**