# Supplementary figures and images for: Deciphering the Metabolic Changes Associated with Diapause Syndrome and Cold Acclimation in the Two-Spotted Spider Mite Tetranychus urticae
Source: PLoS One. 2013 Jan 17;8(1):e54025. doi: 10.1371/journal.pone.0054025 (PMC3547965; doi:10.1371/journal.pone.0054025)

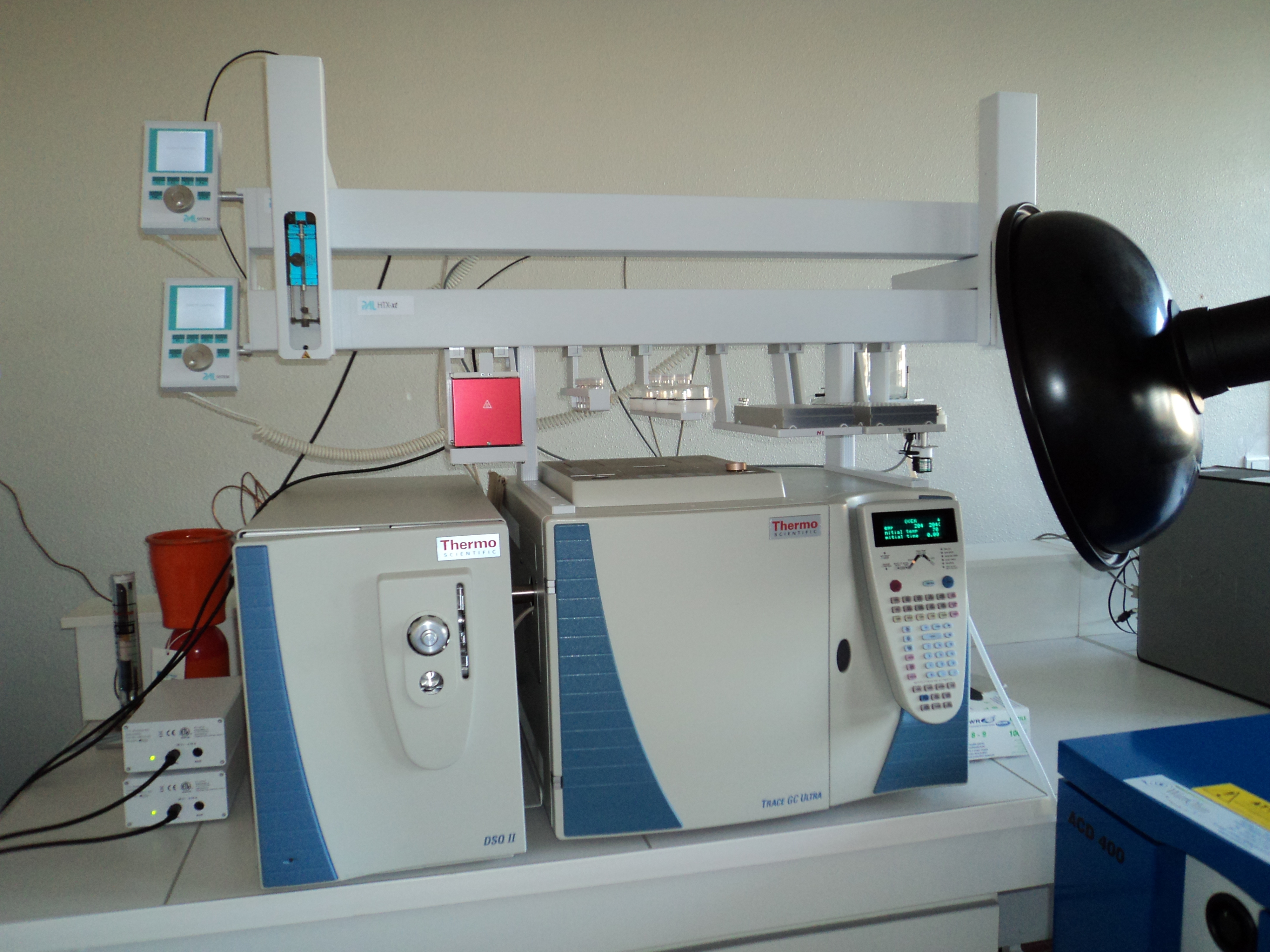

Supplement: Figure S1 — A picture of the GC-MS system., which consists of a Trace GC Ultra chromatograph, a Trace DSQII quadrupole mass spectrometer (Thermo Fischer Scientific Inc, Waltham, MA, USA), and CTC CombiPal autosampler (GERSTEL GmbH and Co.KG, Mülheim an der Ruhr, Germany) that automatized all the derivatization process. (JPG) [file pone.0054025.s001.jpg]
